# Enhanced Thermal Polycondensation of Heavy Coal Tar to Mesophase Pitch via Polyethylene Modification

**DOI:** 10.3390/polym18091027

**Published:** 2026-04-24

**Authors:** Zhengze Huang, Guohua Wang, Hao Shu, Shuaishuai Li, Yang Jia, Yuling Liu

**Affiliations:** 1 School of Physics and Materials Science, Changji University, Changji 831100, China; 18171277001@163.com (Z.H.); wangguohua316@163.com (G.W.); shuhao0518@163.com (H.S.); 2 Xinjiang Key Laboratory of High Value Green Utilization of Low-Rank Coal, Changji University, Changji 831100, China; 3 State Key Laboratory of Eco-Hydraulics in Northwest Arid Region of China, Xi’an University of Technology, Xi’an 710048, China; 1220410003@stu.xaut.edu.cn (S.L.); lyl29992359@163.com (Y.L.)

**Keywords:** heavy coal tar, mesophase pitch, polymer modification, polyethylene, thermal polycondensation

## Abstract

Mesophase pitch (MP) is a high-performance precursor for carbon materials. However, its conventional preparation process is limited by stringent conditions and high costs. In this study, heavy coal tar (HCT) was used as a low-cost carbon source, and polyethylene (PE) was introduced as a modifier to induce MP formation under relatively mild conditions, thereby promoting the thermal polycondensation of HCT. Characterization results show that the addition of different types of PE facilitates the condensation of aromatic molecules and significantly enhances the conversion efficiency of HCT to MP. Among the tested PE types, HDPE exhibits the best performance, with an optimal addition of 6 wt.% at 400 °C, yielding the highest number of uniform mesophase carbon microspheres and the most ordered structure. Based on comprehensive characterization data, an average molecular structure model of the product was constructed, addressing a research gap regarding the role of PE in the thermal polycondensation of HCT. This work provides a new pathway for the energy-efficient preparation and property regulation of MP.

## 1. Introduction

Mesophase pitch (MP), as a key precursor for the preparation of high-performance carbon materials [1], can be converted into advanced materials such as carbon fiber, graphite, and carbon foam through high-temperature carbonization treatment. Among these, carbon fiber [2], with its high specific strength and high specific modulus [3], has become a core strategic material in the aerospace and automotive lightweighting sectors [4]. Graphite, owing to its excellent electrical and thermal conductivity, plays an irreplaceable role in lithium-ion battery anodes and electronic device heat dissipation [5]. Carbon foam, characterized by its ultra-high temperature resistance, demonstrates potential for applications in extreme environments [6]. With the rapid development of strategic emerging industries such as new energy and high-end equipment manufacturing, the global demand for the aforementioned carbon materials is experiencing exponential growth [7]. This renders the research on the controlled synthesis and low-cost preparation technologies of their MP precursors as strategically significant [8].

Since its introduction by Brooks and Taylor in 1961 [9], the theory of carbonaceous mesophase has undergone over six decades of development, forming a systematic theoretical framework, and has been expanded into various application fields. In 1996, Mochida et al., through meticulous observation, proposed the microdomain theory, revealing the existence of oriented microregions within the mesophase and deepening the understanding of its internal organization [10]. In 2004, Wang Chengyang, Li Shuaishuai, and Tong QI, building upon this foundation, proposed the basic structural unit theory [11]. This theory posits that mesophase is assembled from nanoscale spherical basic units through different stacking modes [12], explaining the origin of the multiscale structure of mesophase from a more fundamental level.

At the level of applied research, various strategies have been explored to expand the preparation methods and functionalization pathways of mesophase. Zhang et al. introduced PE during coal pyrolysis, significantly improving the efficiency of coal thermal decomposition [13]. Shimanoe et al. confirmed that hydrogen radicals released during coal heating act as reactive intermediates, promoting the polymerization of small molecules and thereby facilitating mesophase formation [14]. Díez et al. enhanced the quality of the resulting products by adding less than 5% low-density PE (LDPE) and high-density PE (HDPE), demonstrating the potential of polymer blending modification [15]. Guo employed hydrotreatment technology to prepare mesophase with highly ordered microcrystalline structures, laying a material foundation for subsequent applications. In terms of functional applications [16], Zhang et al. utilized MP as an anode material in microbial fuel cells, achieving a high power density of 1800 mW/m^2^, highlighting its potential in energy and environmental fields [17]. Schersche et al. blended MP with PET and spun the mixture into carbon fibers, discovering that the addition of 5% PET significantly enhanced the mechanical properties of the fibers, thereby providing a novel approach for carbon fiber modification [18]. These studies not only deepen the understanding of the formation mechanisms of carbonaceous mesophase but also advance its practical applications across diverse fields such as energy, materials science, and environmental protection.

However, the conventional preparation process for MP requires extremely high temperatures, typically exceeding 2300 °C, to achieve graphitization [19], and it involves complex procedures. In view of this, the present study proposes an additive-induced low-temperature synthesis strategy. Utilizing the heavy fraction of heavy coal tar (HCT), which is rich in aromatic hydrocarbons, aliphatic hydrocarbons, and heterocyclic compounds, as a low-cost carbon source, PE is introduced as a structure-directing agent. During the thermal polycondensation process, PE first melts at approximately 140 °C and uniformly disperses within the HCT matrix, forming an elastic dispersed phase [20]. As the temperature increases, its molecular chains induce the selective polycondensation of aromatic components, generating quinoline insolubles (QI) as nucleation precursors [21]. These QI cores further promote the ordered stacking of surrounding aromatic molecules, leading to the formation of mesophase spheres [22]. Through growth, coalescence, and rearrangement, these spheres ultimately evolve into MP with a highly anisotropic structure [23]. The entire reaction system achieves the construction of ordered molecular structures at temperatures significantly lower than those required by conventional processes, thereby substantially simplifying the preparation procedure [24]. This approach provides a novel strategy for the green and low-cost synthesis of high-performance carbon materials.

This research systematically investigates the structural evolution mechanism of HCT during thermal polycondensation by introducing PE of varying densities as a modifier. A stepwise heat treatment strategy was employed: HCT was first pre-treated with PE of different types to obtain modified heavy coal tar (MHCT), which was subsequently subjected to in-depth heat treatment to produce carbonaceous MP. Fourier-transform infrared spectroscopy (FTIR) was utilized to track the evolution of functional groups in both MHCT and MP. Polarized optical microscopy (POM) was employed to observe the optical anisotropy of MP, with computer vision techniques applied to minimize measurement errors in evaluating mesophase content. X-ray diffraction (XRD) was used to analyze the microcrystalline structural order of MP. Based on these analyses, the influence of PE on the polycondensation reaction pathway of HCT and the formation process of the mesophase structure was systematically elucidated. This work aims to clarify the complete formation mechanism from HCT to MP, thereby filling a gap in the relevant theoretical understanding. This study is expected to pioneer an economical and environmentally friendly route for MP preparation using waste plastics as a co-carbonization agent, providing a theoretical basis for the structural regulation and functional application of MP.

## 2. Materials and Methods

### 2.1. Materials

The HCT used in this study was obtained from a Lantan production company located in Shanxi, China. The PE modifier was sourced from Suzhou Sunjia Plastic Technology Co., Ltd. (Suzhou, China) and Jin Heng Plastic Co., Ltd (Zibo, China). The specific details of the materials are provided in Table S1.

### 2.2. Experimental Methods

**Figure 1:**
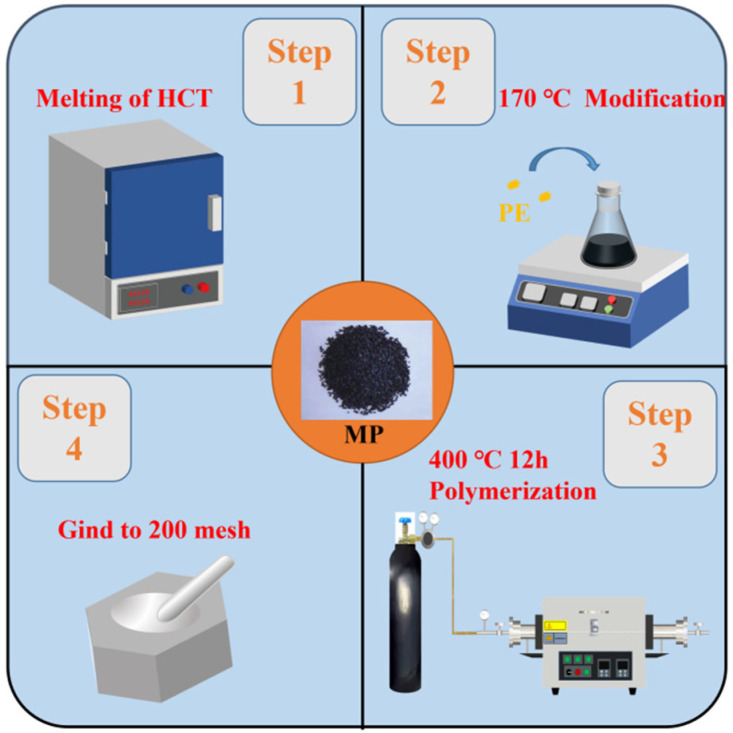
Preparation flowchart.

The preparation process of MP is illustrated in Figure 1. HCT was heated to 170 °C to melt and stirred at 200 rpm for 30 min as a pretreatment. Subsequently, a specific amount of PE polymer was added, and the mixture was stirred at 300 rpm for 1 h to obtain the polymer-modified HCT. The MHCT was then placed in a high-temperature and high-pressure tube furnace (Beike Equipment Technology Co., Ltd, Anhui, China) under a nitrogen atmosphere with the pressure maintained at approximately 0.5 MPa. The reaction was conducted at 400 °C for 12 h, followed by natural cooling to room temperature, yielding the final MP product.

### 2.3. Characterization

The Fourier-transform infrared (FT-IR) spectra were obtained using a VERTEX70 spectrometer (Bruker, Berlin, Germany) with a scanning range of 4000–400 cm^−1^, 28 scans, and a resolution of 0.4 cm^−1^. The ATR mode was applied. A polarized optical microscope (POM) (DM-750P, Leica Microsystems, Wetzlar, Germany) was employed for microscopic observations. X-ray diffraction (XRD) analysis was conducted using an XD-3 diffractometer (Beijing Purkinje General Instrument Co., Ltd., Beijing, China) under the following conditions: scanning range 10–80°, step size 0.02°, Cu Kα radiation with a wavelength of 1.54056 Å, operating voltage 36 kV, and current 20 mA. The ^13^C-nuclear magnetic resonance (^13^C-NMR) spectra were recorded on a Bruker 700M spectrometer, with deuterated chloroform (CDCl_3_) as the solvent. Gel permeation chromatography (GPC) was performed using a Shimadzu RID-20A system with a sample concentration of approximately 20 mg/mL. The system was equipped with an LC20 high-performance liquid chromatography pump (Shimadzu, Japan) and an RID-20 refractive index detector (Shimadzu, Kyoto, Japan). Elemental analysis (ultimate analysis, UA) was conducted using an Elementar Unicube analyzer (Elementar Analysensysteme GmbH, Langenselbold, Germany) to determine the elemental composition of the MHCT and its MP. The CHNS mode was applied, and the oxygen content was obtained by difference.

### 2.4. Microcrystalline Structure Analysis

The data obtained from testing were analyzed using the Jade software (Jade 9.0), along with the Powder Diffraction File (PDF) card database, to compare three parameters of the material: interplanar spacing, intensity ratio, and Miller indices (*d* − *I/I*_0_ − (*hkl*)) [25]. The Bragg equation (Equation (1)), Scherrer’s formula (Equation (2)), and Equations (3) and (4) were then used to calculate the unit cell parameters of MP [26].

(1) $$d_{002}=\frac{\lambda }{2\cdot \cos\theta }$$

(2) $$L_{c}=\frac{0.89\lambda }{\beta \cdot \cos\theta }$$

(3) $$N=\frac{Lc}{d_{002}}+1$$

(4) $$n=0.32N^{2}$$

where *d*_002_ represents the interlayer spacing of carbon microcrystals (nm); *L_C_* is the crystal grain thickness (nm); *λ* is the X-ray wavelength; *β* is the full width at half maximum of the diffraction peak corresponding to the Miller index (*hkl*); *θ* is the diffraction angle of the (*hkl*) peak (°); *N* is the number of aromatic carbon layers; and n is the number of aromatic carbon atoms per layer.

## 3. Results and Discussion

### 3.1. Different Types of PE on MP Formation

The modification of HCT with PE for the preparation of MP significantly promotes the formation of mesophase spheres, an effect that likely extends to the MP itself. The aggregation of MP typically evolves during formation, initially manifesting as dispersed small domains [8]. Consequently, the optical texture observed under POM appears fragmented and has not yet reached a unified structure. Previous studies have indicated that in feedstocks containing QI [27], these insolubles serve as the core for the formation of mesophase carbon microspheres during thermal polymerization. Through the formation, growth, coalescence, and rearrangement of these mesophase spheres, MP is ultimately produced [28].

**Figure 2:**
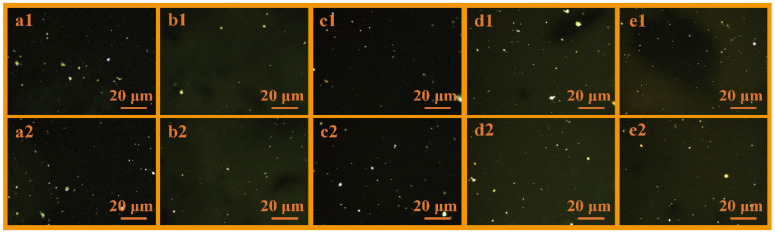
POM images of mesophase structures modified by different types of PE: (**a1**,**a2**) MP-HDPE; (**b1**,**b2**) MP-MDPE; (**c1**,**c2**) MP-LDPE; (**d1**,**d2**) MP-LLDPE; (**e1**,**e2**) MP-ULDPE.

Figure 2 presents the POM images of MP modified with five types of PE, denoted as MP-HDPE, MP-MDPE, MP-LDPE, MP-LLDPE, and MP-ULDPE. Compared to unmodified HCT, all types of PE significantly promote the formation of mesophase carbon microspheres by enhancing the coalescence process. This leads to an increase in both the volume fraction of the mesophase region and the particle size distribution of the mesospheres. This enhancement is attributed to the regulation of the complex hydrocarbon system by hydrogen radicals generated from PE pyrolysis, which facilitates the ordered stacking of planar aromatic molecules in the liquid phase and promotes nucleation through interaction with QI [29]. Consequently, the polycondensation of polycyclic aromatic hydrocarbons is optimized. Notably, the MP-HDPE system exhibits the most uniform mesophase distribution and is enriched with larger-sized carbon microspheres.

Further analysis was conducted using computer vision techniques to obtain the area and diameter data of the mesophase polarized images in Figure 2, as presented in Figures S1 and S2. The HDPE-modified sample exhibits the largest total area (10,597 μm^2^) and relatively large sphere diameters (125 μm), indicating its superior ability to simultaneously promote both the nucleation and growth of mesophase spheres. Although the LDPE and LLDPE systems generate relatively large individual spheres, their overall mesophase volume is lower than that of the HDPE system, while the ULDPE system shows the smallest particle size. Based on quantitative analysis data, the effectiveness of PE types in promoting mesophase sphere formation can be ranked as follows: HDPE > MDPE ≈ LLDPE > LDPE > ULDPE. Therefore, HDPE was selected as the optimal modifier for subsequent experiments.

### 3.2. Different Dosages of HDPE on MP Formation

**Figure 3:**
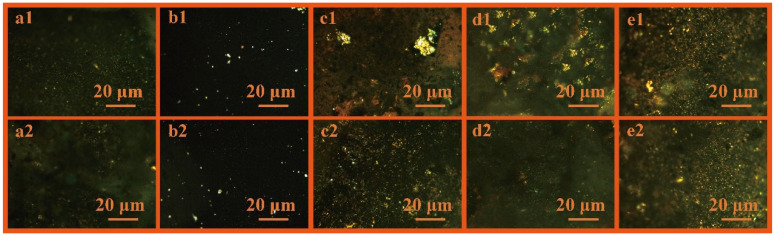
POM images of mesophase structures at different HDPE dosages: (**a1**,**a2**) MP-HDPE-2%; (**b1**,**b2**) MP-HDPE-4%; (**c1**,**c2**) MP-HDPE-6%; (**d1**,**d2**) MP-HDPE-8%; (**e1**,**e2**) MP-HDPE-10%.

**Figure 4:**
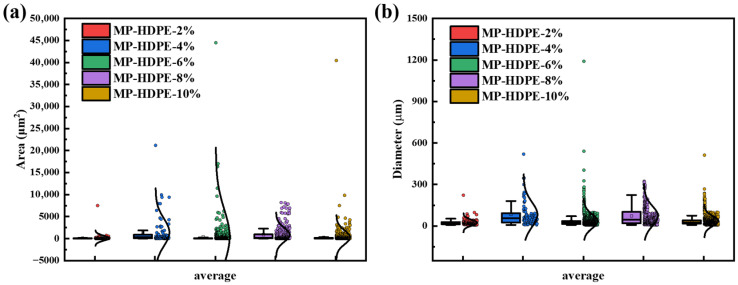
(**a**) Mesophase average diameter at different HDPE dosages; (**b**) mesophase average area at different HDPE dosages.

Figure 3 presents the POM images of MHCT modified with five different dosages of HDPE, designated as MP-HDPE-2%, MP-HDPE-4%, MP-HDPE-6%, MP-HDPE-8%, and MP-HDPE-10%. Using computer vision techniques, the area and diameter data of the mesophase regions were extracted from the POM images in Figure 3 and plotted in Figures S3 and S4, with the numbering corresponding to the images in Figure 3. The extracted data were then plotted, as shown in Figure 4. From Figure 4a, it can be observed that the MP-HDPE-6% sample exhibits a maximum mesophase area of 46,005 μm^2^, which is significantly higher than that of samples prepared with other dosages. Meanwhile, Figure 4b shows that the diameter of mesophase carbon microspheres does not differ substantially between the 6% and 8% addition levels. However, the 6% addition yields the most uniform size distribution and the highest number of mesophase carbon microspheres, indicating good dispersion and fluidity, accompanied by a substantial increase in the number of mesophase carbon microspheres (see Table S2 for detailed data).

This enhancement is attributed to the appropriate amount of HDPE, which not only supplies hydrogen radicals but also provides suitable nucleation sites by replacing heteroatoms, forming QI-like species that promote the polycondensation of aromatic molecules. An insufficient addition of HDPE leads to inadequate nucleation sites, resulting in an insufficient quantity of QI and thus limiting mesophase formation [30]. Conversely, excessive addition may cause over-absorption of light components by PE or insufficient decomposition, increasing system viscosity and hindering the growth and coalescence of mesophase spheres [29]. Therefore, a PE addition of 6 wt.% achieves an optimal balance between promoting polycondensation reactions, enhancing mesophase uniformity, and controlling mesophase formation.

**Figure 5:**
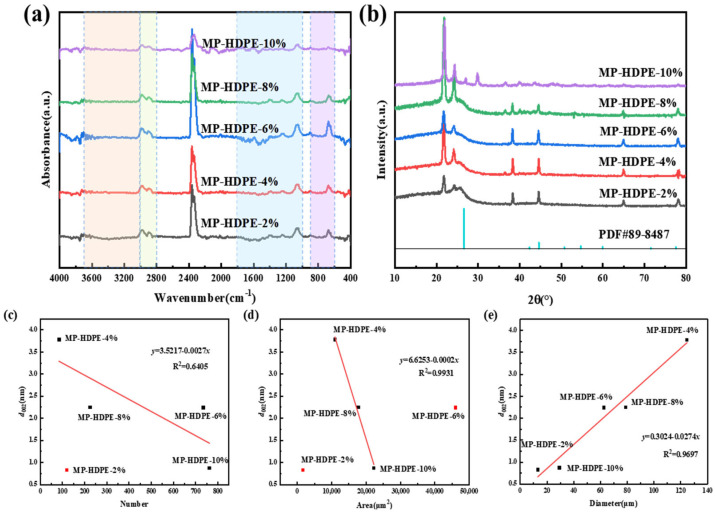
(**a**) FTIR. The orange region (3700–3100 cm^−1^) is the -OH region, the yellow region (3000–2800 cm^−1^) is the stretching vibration region of aliphatic hydrocarbons C-H, the blue region (1800–1200 cm^−1^) is the stretching vibration region of C-C single bonds such as ether bonds, carbonyl groups, and carboxyl groups, and the purple region (900–600 cm^−1^) is the stretching vibration region of aromatic hydrocarbons C-H; (**b**) XRD; (**c**) number of mesophase spheres and carbon microcrystal interlayer spacing; (**d**) average total mesophase area and carbon microcrystal interlayer spacing; (**e**) average mesophase diameter and carbon microcrystal interlayer spacing.

Figure 5a presents the FTIR of MP with varying HDPE additions. As the HDPE content increases, the aromatic functional group peaks initially intensify, reaching a maximum at 6 wt.%. Beyond this threshold, the peak intensity decreases, and at 10 wt.%, the aromatic peaks nearly disappear, indicating that excessive HDPE addition inhibits MP formation, consistent with the previous POM analysis. Figure 5b shows the XRD patterns of MP with different HDPE dosages. Previous studies have demonstrated that a higher proportion of the graphitic carbon peak at approximately 26° facilitates layered molecular stacking and the formation of ordered structures, yielding high-performance MP [9]. In contrast, an increase in the amorphous carbon peak at around 23° is detrimental to the formation of graphitic structures. Among the samples, MP-HDPE-10% exhibits the most impurity peaks and the poorest crystallinity, whereas MP-HDPE-6% shows the smallest amorphous carbon peak at 23°, consistent with the superior uniformity observed in POM for MP-HDPE-6%. Therefore, the optimal modification condition for MP preparation is determined to be 6 wt.% HDPE addition.

The microcrystalline structural parameters calculated using Equations (1)–(4) (Table S3) reveal that MP-HDPE-6% possesses a smaller interlayer spacing (*d*_002_) and a larger stacking height (*L_C_*). Previous research has reported that MP derived from HCT exhibits *d*_002_ spacing, stacking height (*L_C_*), number of carbon layers (*N*), and number of carbon atoms per layer (n) of 0.89, 1.86, 3.11 [31], and 3.09, respectively. To elucidate the relationship between mesophase content and graphite microcrystalline structure, correlations between mesophase parameters and carbon microcrystalline interlayer spacing for PE-modified HCT-based MP were established, as shown in Figure 5c–e. Correlation analysis further reveals a significant negative correlation between mesophase area and *d*_002_ (R^2^ = 0.9931), while mesophase diameter shows a significant positive correlation with *d*_002_ (R^2^ = 0.9697).

### 3.3. Chemical Analysis of MP Formation with 6 wt.% HDPE

The elemental analysis results for MP-HDPE-6% are presented in Table 1. Based on the elemental content, the atomic ratios of each element in the modified HCT were calculated, yielding the empirical formula C_7.10_H_5.73_N_0.08_S_0.00_O_0.47_ for MP-HDPE-6%.

**Figure 6:**
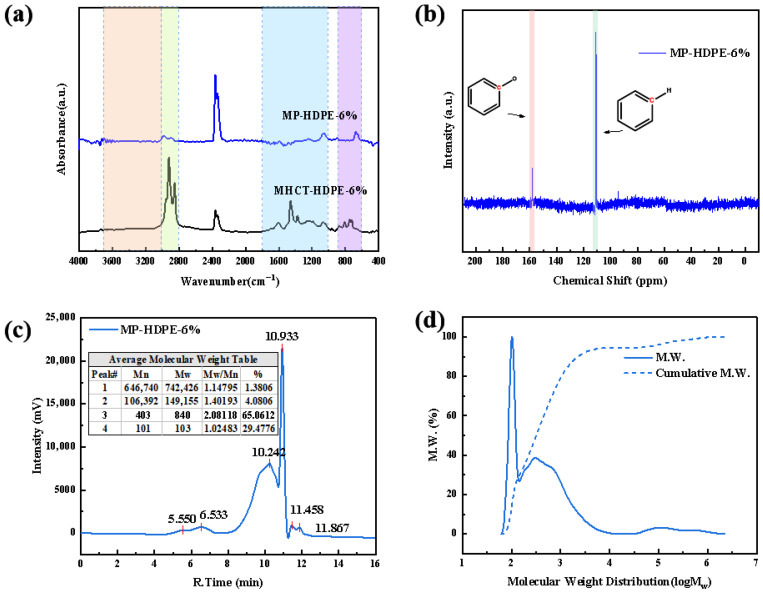
(**a**) FTIR; (**b**) ^13^C-NMR; (**c**) GPC; (**d**) molecular weight distribution curve of MP-HDPE-6%.

Figure 6a presents the FTIR spectra of MHCT and MP after treatment. In the hydroxyl region (3700–3100 cm^−1^), the O–H vibration peak of water molecules at approximately 3300 cm^−1^ disappears in MHCT-HDPE-6%, indicating complete removal of water from the treated sample. It can be inferred that the hydroxyl structure region in MP-HDPE-6% primarily arises from O-H stretching vibrations of phenols and alcohols [32]. In the aliphatic C-H stretching vibration region (3000–2800 cm^−1^), the peak intensity of MP-HDPE-6% is significantly reduced, suggesting an increased degree of internal condensation [33]. The CO_2_ absorption peak near 2400 cm^−1^ originates from reactions involving small molecular fragments generated by the pyrolysis of the modified coal tar matrix and HDPE molecules, as well as oxygen-containing functional groups; consequently, this peak is markedly stronger in MP than in MHCT. In the C-C single bond stretching vibration region (1800–1000 cm^−1^), which includes ether, carbonyl, and carboxyl groups, the intensity of carbonyl and hydroxyl functional groups decreases. This is attributed to intramolecular dehydration occurring in MHCT at 400 °C, leading to the formation of ether bonds and CO_2_. In the aromatic C-H out-of-plane bending vibration region (900–600 cm^−1^), combined with the significantly stronger characteristic C=C skeleton vibration peaks of aromatic carbons compared to aliphatic C-H in MP, a higher degree of polycondensation of fused aromatic hydrocarbons is indicated, which facilitates the coalescence process of the MP [34].

Figure 6b shows the ^13^C-NMR spectrum, further confirming that the signal for oxygen-substituted aromatic carbons near 158 ppm is lower than that for protonated aromatic carbons around 110 ppm. Consistent with the previous FTIR analysis [35], these findings suggest that the molecular framework of MP-HDPE-6% primarily consists of polycyclic aromatic hydrocarbon structures, with a small number of residual structural units possibly connected through phenolic hydroxyl groups or ether linkages. The polycyclic aromatic rings exhibit high compatibility due to strong π–π stacking interactions. Furthermore, the protonated aromatic hydrogens can form hydrogen bonds with oxygen-containing functional groups, which contribute to the fluidity and dispersibility of MP. These synergistic interactions further promote the formation of a more compact and ordered structure within MP.

**Figure 7:**
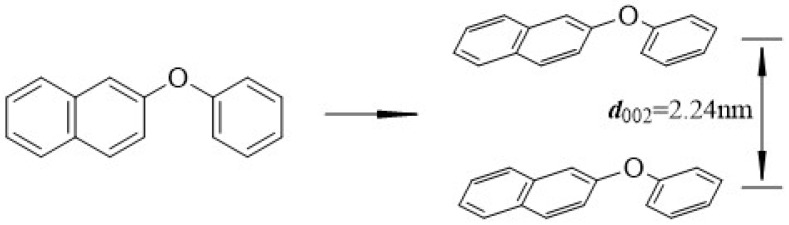
Chemical structure and interlayer structure of carbon microcrystals on MP-HDPE-6%.

GPC analysis in Figure 6c reveals that, generally, larger molecular species elute earlier. Based on GPC principles, MP-HDPE-6% exhibits two main peaks at 10.242 min and 10.933 min, corresponding to asphaltenes and resins and aromatic and saturated components, respectively, accounting for 65.06% and 29.48% of the total peak area. Figure 6d displays the molecular weight (Mw) distribution curve of MP-HDPE-6%. The relative Mw is primarily concentrated in the range of 10^1.5^–10^4^ Da, with a mesophase carbon peak observed around 10^4.5^ to 10^6.5^ Da. Table S4 summarizes the average Mw data obtained from GPC. Based on elemental analysis and the empirical formula of MP-HDPE-6%, the average number of molecular units was calculated using the number-average molecular weight (Mn). The molecular formula was derived by multiplying the unit count by the empirical formula, yielding C_15.67_H_12.64_N_0.18_S_0.01_O_1.04_ for MP-HDPE-6% [36]. Rounding these values gives the molecular formula C_16_H_13_O. Combining this with the ^13^C-NMR results and the previously discussed carbon microcrystalline interlayer spacing, a stacking model for MP-HDPE-6% is constructed, as shown in Figure 7.

The formation of mesophase is anchored in the ordered stacking of planar aromatic molecules within the isotropic liquid phase of HCT. This study reveals that the superior efficacy of HDPE in inducing mesophase formation among various PE additives stems from its unique molecular structure and pyrolysis behavior. HDPE, characterized by high crystallinity and highly regular linear molecular chains, exhibits a more ordered and controllable pyrolysis process within the thermal polycondensation system. Firstly, HDPE pyrolysis generates an optimal quantity of hydrogen radicals, which effectively quench the active radical sites at the edges of polycyclic aromatic hydrocarbon molecules in coal tar [37]. This inhibits premature and disordered intermolecular cross-linking polycondensation, thereby maintaining the fluidity of the reaction system and providing the kinetic conditions necessary for the migration and ordered stacking of aromatic molecules. Secondly, the olefin and small alkyl fragments released during HDPE pyrolysis can alkylate aromatic molecules; these alkyl side chains subsequently undergo dehydrogenation, cyclization [38], and other reactions at elevated temperatures, acting as intermolecular “bridges” or “stitches.” This promotes the formation of planar aromatic macromolecules with suitable size and reactivity, providing structural units for mesophase development. Under this dual regulation, driven thermodynamically by liquid crystal formation, the system develops large, well-perfected mesophase spheres through π–π stacking. These spheres ultimately coalesce to form the well-developed, broad-area texture observed in POM, corresponding to the higher microcrystalline order revealed by XRD. This characteristic of “modulated regulation and controlled release” during pyrolysis renders HDPE significantly superior to other PE types in promoting ordered mesophase development.

## 4. Conclusions

This study investigated the preparation of MP by modifying HCT with different types and addition amounts of PE, revealing the critical role of PE in regulating mesophase formation and promoting the growth and ordered stacking of mesophase spheres. The experimental results demonstrate that PE, through thermal decomposition, releases hydrogen radicals and small molecular fragments, effectively modulating the reaction behavior of polycyclic aromatic hydrocarbons and promoting their ordered polycondensation and stacking. This significantly facilitates the formation and growth process of mesophase spheres. Specifically, a PE addition amount of 6 wt.% yields the largest mesophase area and the most uniform distribution, whereas excessive or insufficient PE addition disrupts the reaction equilibrium of the system, affecting mesophase formation and structural integrity. FTIR and XRD analyses further confirm that at this addition level, the content of aromatic functional groups is highest, and the graphitic carbon structure is prominent. The calculated microcrystalline structural parameters indicate superior ordering and crystallinity.

The optimal sample, MP-HDPE-6%, was further characterized in detail using elemental analysis, FTIR, ^13^C-NMR, and GPC. The results reveal that this MP possesses a high degree of condensation, with a molecular framework predominantly composed of polycyclic fused aromatic structures and a low abundance of functional groups such as hydroxyl and carbonyl, reflecting a favorable thermal polycondensation process. GPC analysis further validated the structural orderliness and stability. Based on elemental analysis and number-average molecular weight, the molecular formula was derived as C_16_H_13_O, and a stacking structure model was constructed in conjunction with spectroscopic data.

In conclusion, this study establishes HDPE as the optimal modifier and 6 wt.% as the optimal addition amount. It elucidates the modification mechanism by which HDPE suppresses disordered polycondensation and promotes the ordered stacking of aromatic hydrocarbons by providing an active hydrogen source. These findings provide a theoretical basis and experimental support for the efficient preparation of high-quality MP.

This also lays a foundation for the future synthesis of carbon materials from waste plastics and heavy coal tar. Introducing additional functional groups, particularly during the PE modification process, may enhance the surface activity of the resulting material, thereby improving its performance in the synthesis of carbon materials.

## Figures and Tables

**Table 1:** Elemental composition of MP-HDPE-6%.

Sample	C	H	N	S	O	H/C Radio
MP-HDPE-6%	85.31	5.78	1.19	0.13	7.59	0.014

## Data Availability

The original contributions presented in this study are included in the article/Supplementary Material. Further inquiries can be directed to the corresponding author.

## Supplementary Materials

The following supporting information can be downloaded at: https://www.mdpi.com/article/10.3390/polym18091027/s1. Table S1: Parameters of Different Types of PE Polymers; Table S2: Mesophase parameters of MP modified by different additive dosages of HDPE; Table S3: XRD structure parameters of different additions of PE modified MP; Table S4: The GPC average molecular weight; Figure S1: Mesophase area modified by different types of PE. (a1-a2) MP-HDPE; (b1-b2) MP-MDPE; (c1-c2) MP-LDPE; (d1-d2) MP-LLDPE; (e1-e2) MP-ULDPE; Figure S2: Mesophase sphere diameters modified by different types of PE. (a1-a2) MP-HDPE; (b1-b2) MP-MDPE; (c1-c2) MP-LDPE; (d1-d2) MP-LLDPE; (e1-e2) MP-ULDPE; Figure S3: Mesophase sphere diameters at different HDPE dosages. (a1-a2) MP-HDPE-2%; (b1-b2) MP-HDPE-4%; (c1-c2) MP-HDPE-6%; (d1-d2) MP-HDPE-8%; (e1-e2) MP-HDPE-10%; Figure S4: Mesophase sphere diameters at different HDPE dosages. (a1-a2) MP-HDPE-2%; (b1-b2) MP-HDPE-4%; (c1-c2) MP-HDPE-6%; (d1-d2) MP-HDPE-8%; (e1-e2) MP-HDPE-10%.
