# Supplementary material for: Enhanced Thermal Polycondensation of Heavy Coal Tar to Mesophase Pitch via Polyethylene Modification
Source: Polymers (Basel). 2026 Apr 24;18(9):1027. doi: 10.3390/polym18091027 (PMC13165213; doi:10.3390/polym18091027)
Supplement: Supplementary file 1 [file polymers-18-01027-s001.zip › polymers-4253415-supplementary.pdf]

**Table S1 Parameters of Different Types of PE Polymers**

| Type | Density（$g/cm^3$） | Crystallinity（%） | Chemical formula |
|---|---|---|---|
| HDPE（High Density Polyethylene） | 0.950 | 85~95 | $(CH_2CH_2)_x$ |
| MDPE（Medium Density Polyethylene） | 0.935 | 70~85 | |
| LDPE（Low Density Polyethylene） | 0.920 | 65~75 | |
| LLDPE（Linear Low Density Polyethylene） | 0.918 | 55~65 | |
| ULDPE（Ultra Low Density Polyethylene） | 0.905 | 20~55 | |

**Table S2 Mesophase parameters of MP modified by different additive dosages of HDPE**

| simple | number | area（$\mu m^2$） | diameter（μm） |
|---|---|---|---|
| MP-HDPE-2% | 120 | 1591 | 13.26 |
| MP-HDPE-4% | 85 | 10597 | 124.67 |
| MP-HDPE-6% | 735 | 46005 | 62.59 |
| MP-HDPE-8% | 225 | 17733 | 78.81 |
| MP-HDPE-10% | 762 | 22280 | 29.24 |

**Table S3 XRD structure parameters of different additions of PE modified MP**

| simple | $2\theta_{002}$/(°) | $\theta_{002}$/(°) | β | $d_{002}$/nm | Lc/nm | N | n |
|---|---|---|---|---|---|---|---|
| MP-HDPE-2% | 24.35 | 12.18 | 5.88 | 0.83 | 1.99 | 3.83 | 4.69 |
| MP-HDPE-4% | 22.40 | 11.20 | 9.59 | 3.78 | 5.10 | 9.89 | 31.29 |
| MP-HDPE-6% | 22.69 | 11.35 | 10.67 | 2.24 | 2.75 | 5.98 | 11.46 |
| MP-HDPE-8% | 22.69 | 11.35 | 9.29 | 2.25 | 3.17 | 6.42 | 13.20 |
| MP-HDPE-10% | 24.16 | 12.08 | 2.16 | 0.87 | 5.63 | 7.50 | 18.01 |

**Table S4 The GPC average molecular weight**

| simple | Mn | Mw | Mz | Mzl | Mw/Mn | Mz/Mw |
|---|---|---|---|---|---|---|
| MP-HDPE-6% | 220 | 16913 | 600324 | 930057 | 76.78812 | 35.49487 |

Number Average Molecular Weight（Mn）：The average molecular mass calculated based on the number of molecules.

Weight Average Molecular Weight（Mw）：The average molecular weight calculated based on the mass ratio.

z-Average Molecular Weight（Mz）：The average molecular weight calculated based on the third power of mass.

z1-Average Molecular Weight（Mz1）：The average molecular weight calculated based on the fourth power of mass.

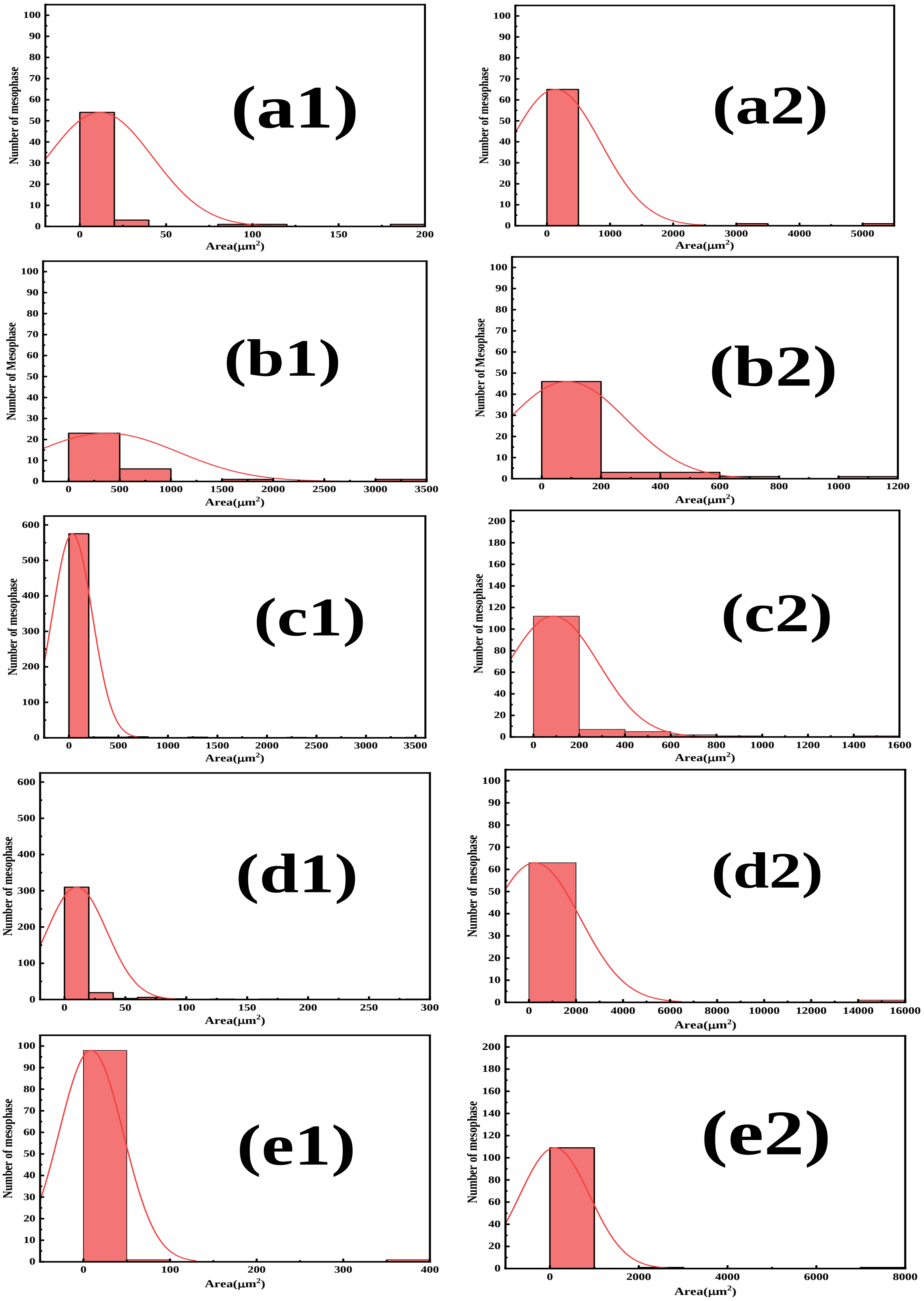


**Fig. S1 Mesophase area modified by different types of PE**

（a1-a2）MP-HDPE;（b1-b2）MP-MDPE;（c1-c2）MP-LDPE;

（d1-d2）MP-LLDPE;（e1-e2）MP-ULDPE

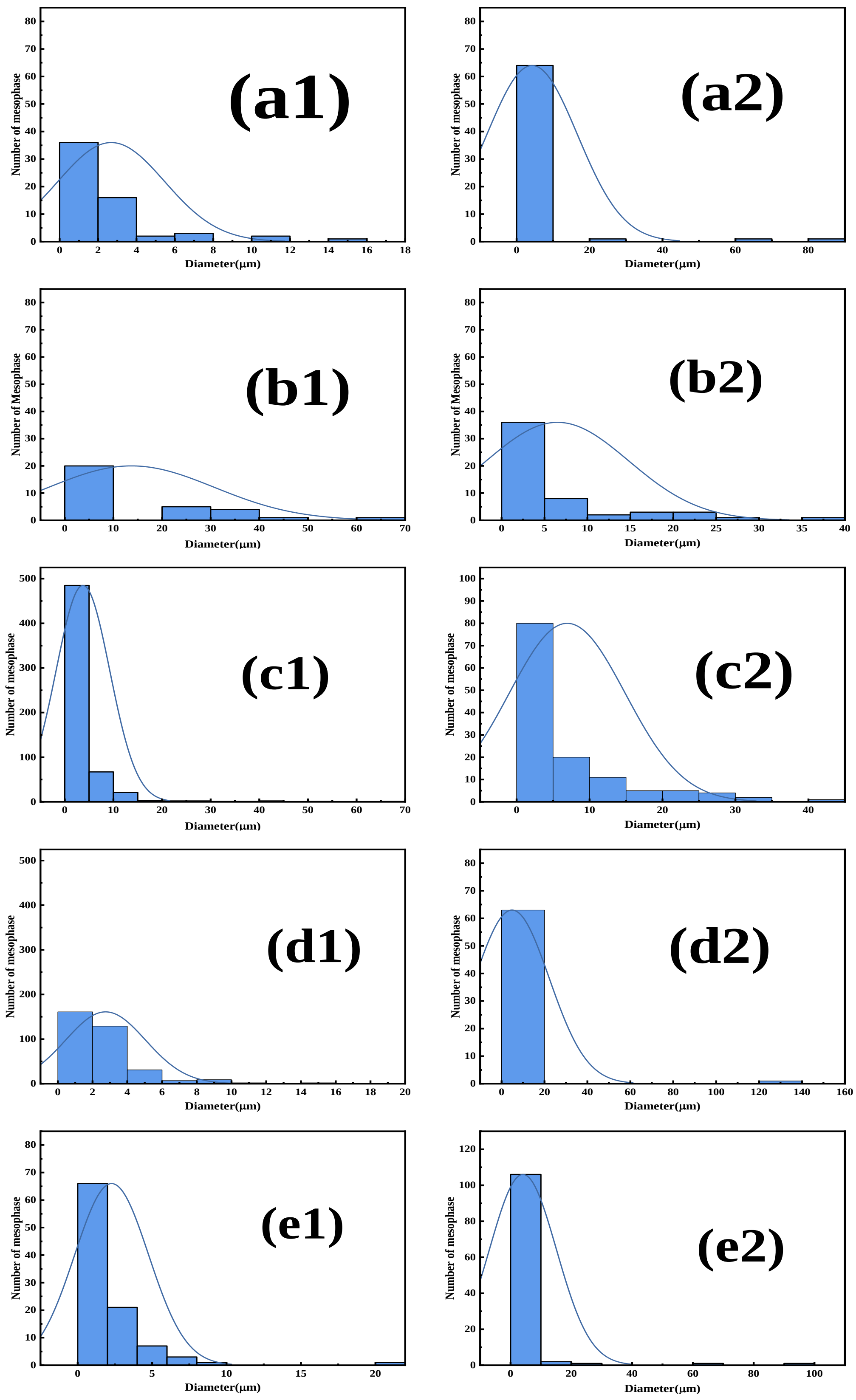


**Fig. S2 Mesophase sphere diameters modified by different types of PE**

（a1-a2）MP-HDPE;（b1-b2）MP-MDPE;（c1-c2）MP-LDPE;

（d1-d2）MP-LLDPE;（e1-e2）MP-ULDPE

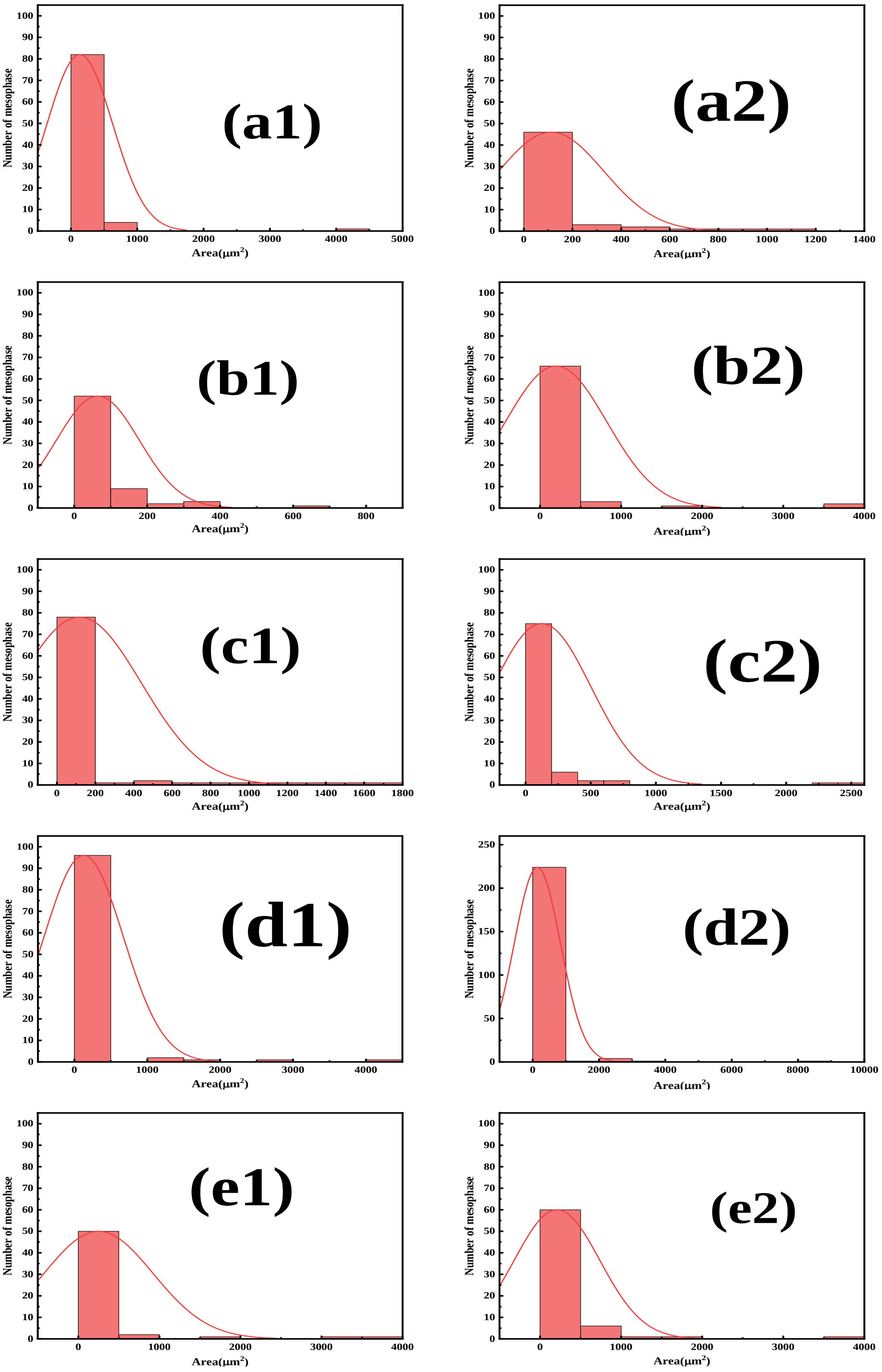


**Fig. S3 Mesophase sphere diameters at different HDPE dosages**

（a1-a2）MP-HDPE-2%;（b1-b2）MP-HDPE-4%;（c1-c2）MP-HDPE-6%;

（d1-d2）MP-HDPE-8%;（e1-e2）MP-HDPE-10%.

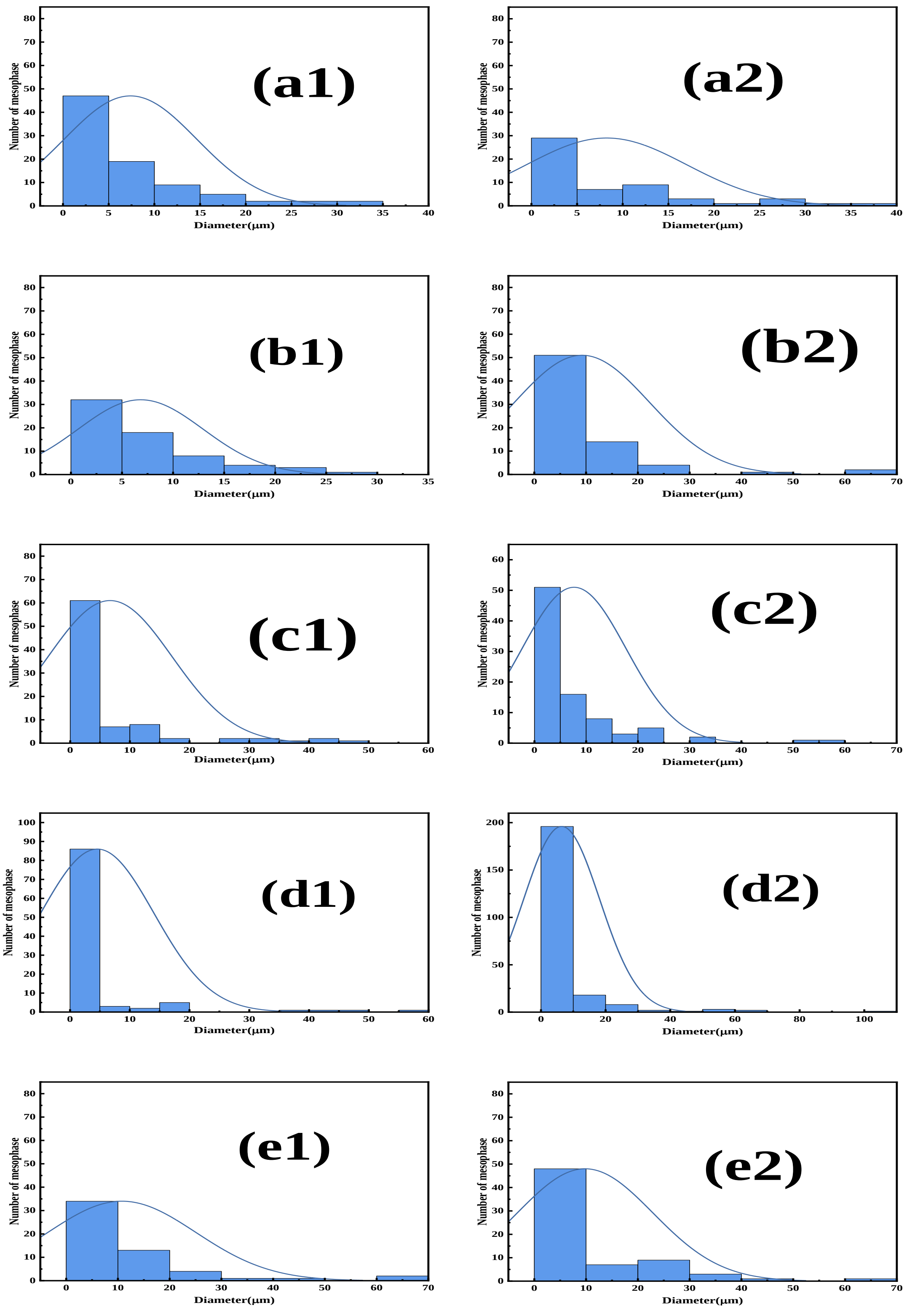


**Fig. S4 Mesophase sphere diameters at different HDPE dosages**

（a1-a2）MP-HDPE-2%;（b1-b2）MP-HDPE-4%;（c1-c2）MP-HDPE-6%;

（d1-d2）MP-HDPE-8%;（e1-e2）MP-HDPE-10%.
